# Digital Health Literacy and Attitudes Toward eHealth Technologies Among Patients With Cardiovascular Disease and Their Implications for Secondary Prevention: Survey Study

**DOI:** 10.2196/63057

**Published:** 2025-03-19

**Authors:** Greta Ullrich, Alexander Bäuerle, Hannah Vogt, Amir Abbas Mahabadi, Katrin Paldán, Daniel Messiha, Lisa Maria Jahre, Christos Rammos, Tienush Rassaf, Julia Lortz

**Affiliations:** 1 Department of Cardiology and Vascular Medicine, West-German Heart and Vascular Center Essen, University of Duisburg-Essen, Essen, Germany; 2 Clinic for Psychosomatic Medicine and Psychotherapy, LVR-University Hospital Essen, Essen, Germany; 3 Center for Translational Neuro- and Behavioral Sciences (C-TNBS), University of Duisburg-Essen, Essen, Germany; 4 HCT Research, Vorarlberg University of Applied Science, Dornbirn, Austria

**Keywords:** cardiovascular diseases, telemedicine, eHealth, patient-centered approach, digital health literacy, digital health, cardiovascular disease, mortality, artery disease, ischemic, heart disease, diabetes mellitus, obesity, patient education, eHealth literacy, mobile phone

## Abstract

**Background:**

Cardiovascular disease is the major cause of death worldwide, leading to a significant socioeconomic burden. Although secondary prevention is a cornerstone in chronic disease management, adherence to guideline recommendations in this regard often falters, leading to suboptimal outcomes. While eHealth technologies are promising for improving treatment adherence, they also represent a new approach to secondary prevention. However, a common critique is that extensive digitalization may not adequately address the needs of older adults with chronic medical conditions.

**Objective:**

This study aims to analyze eHealth literacy, digital use patterns, and general attitudes toward digital technologies in a collective of patients with cardiovascular disease to identify potential obstacles in implementing mobile health technologies in secondary preventive therapy.

**Methods:**

This survey-based study was a part of the baseline examination of the PreventiPlaque trial. It involved 240 participants with known coronary artery disease. The assessment evaluated their current understanding of the general use of digital devices. The questionnaire covered aspects such as the duration of daily use, personal attitudes, and the perceived burden associated with digital media. eHealth literacy was assessed within the target population and general demographic data were gathered, focusing on cardiovascular comorbidities and risk factors.

**Results:**

The analysis revealed an average age of 61.9 (SD 8.9) years, with 59.9% (n=144) of the participants being male. Overall, 37.3% (n=90) of the participants had previous knowledge of digital health interventions, while only 17.8% (n=41) had used them. Despite the generally low practical application within this study population, there was a high level of confidence in handling digital devices, with 61.9% (n=149) expressing themselves as either rather confident or very confident. Regarding the levels of eHealth literacy among the participants, 71.2% (n=170) claimed to be familiar with locating health information on the internet, and 64% (n=153) of participants felt capable of critically evaluating its quality. These levels of digital confidence were consistent across all age groups. Moreover, internet use rates remained high even among the older participants, with 80% (n=192) of those participants older than 75 years using the internet for 1-3 hours a day.

**Conclusions:**

The study unveiled a notable confidence level among participants regarding the use of digital devices, coupled with a favorable attitude toward digital media evident across all age brackets. Remarkably, internet use rates remained high, even among older participants. The actual utilization of digital health interventions was relatively low, potentially stemming from challenges in locating reliable sources. These findings emphasize the prospect of future eHealth interventions customized to the distinct needs and preferences of patients in cardiovascular disease management. Recognizing the incongruity between confidence in device use and the restricted adoption of digital health tools can guide the development of focused interventions to narrow this divide.

## Introduction

Cardiovascular disease remains the leading cause of global mortality, with coronary artery disease and ischemic heart disease alone contributing to 16% of total annual deaths worldwide [1]. Over the past 2 decades, ischemic heart disease has demonstrated the most rapid growth in overall death rates globally, resulting in nearly 9 million annual deaths [1]. Cardiovascular disease is intricately linked to various comorbidities and a severe mental health burden [2]. While advancements in the understanding and treatment of cardiovascular disease persist, there is an increasing emphasis on secondary prevention. Following guideline recommendations, secondary prevention aims to address modifiable risk factors such as hyperlipidemia, nicotine consumption, arterial hypertension, diabetes mellitus, obesity, chronic stress, and lack of physical activity [3]. Despite the preventive potential of lifestyle modifications supported by guidelines, their implementation remains inadequate. Primary obstacles include the challenge of ensuring adherence to long-term behavioral changes and the scarcity of medical resources and time needed for comprehensive patient education in chronic disease management [4,5]. Digital health interventions emerge as a promising avenue to educate and empower patients, encouraging an active role in disease management while optimizing the control and monitoring of modifiable risk factors [6]. They offer a potential solution to the current deficiencies in medical infrastructure, addressing the escalating demand associated with the increasing prevalence of cardiovascular disease [7]. However, a frequently cited challenge in the expanding digitization of health care is the consideration of special needs among older patients concerning the development and structure of digital health interventions [8]. Given that cardiovascular disease predominantly affects older individuals, it is crucial to not disadvantage them to fully use the modern treatment options.

The objective of this study was to delve into the digital use patterns of patients with cardiovascular disease expressing an interest in digital health interventions. The aim was to assess and identify potential obstacles and challenges in the implementation of mobile health (mHealth) or eHealth technologies for secondary prevention of cardiovascular disease. The study sought to evaluate patients’ existing knowledge and opinions regarding smartphones, internet, and digital health interventions while focusing on uncovering possible age-dependent differences. The overarching goal was to gain insights that would inform the design of digital health interventions tailored to meet the specific needs and requirements of individuals managing cardiovascular conditions.

## Methods

### Study Design and Participants

We conducted a survey-based assessment at the University Hospital Essen, West German Heart and Vascular Center, Department of Cardiology and Vascular Medicine. The questionnaires used were part of the baseline examination of the PreventiPlaque trial. PreventiPlaque is a registered randomized clinical trial (NCT05096637) testing the effects of a smartphone app that included atherosclerotic plaque visualization on adherence to secondary preventive therapy [9]. The recruitment of participants for the PreventiPlaque trial occurred in 2022. Patients with atherosclerotic cardiovascular disease were eligible for participation in this trial. This included patients with documented ischemic heart disease, acute coronary syndrome, and patients with proven peripheral artery disease [10]. Another requirement was adult age (18 years and older). Participants had to own a smartphone that was suitable for potential app use. Finally, patients had to give written informed consent to comply with the study protocol and had to be willing to participate in the study. Patients with insufficient knowledge of the German language, or unwillingness to use a smartphone app, were excluded from the study.

### Ethical Considerations

The conduct of the trial was approved by the ethics committee of the Medical Faculty of the University of Duisburg-Essen (20‐9157-BO). Written informed consent was provided by each participant before inclusion in the study. The study was conducted in accordance to the Declaration of Helsinki. Data were collected and analyzed using a pseudonymous form. The participants did not receive any form of financial compensation for participating in this trial.

### Measurements

#### Sociodemographic and Medical Data

Basic sociodemographic data and medical data were collected, using a questionnaire with 15 items, including the patient’s marital status, level of education, and current profession. Moreover, patients were asked to self-assess their level of physical activity, as well as the quality of their diet. We extracted medical information from the hospital’s electronic medical records regarding the presence of major cardiovascular risk factors such as diabetes mellitus, arterial hypertension, and hypercholesterolemia, as well as the prevalence of cardiovascular comorbidities including coronary artery disease, peripheral artery disease, stroke, aortic syndrome, or chronic heart failure.

#### eHealth Literacy

The revised German eHealth Literacy Scale (GR-eHEALS) was used to assess the participants’ skills to find and critically evaluate health information on the internet [11]. It is a validated tool to measure eHealth literacy in patients with cardiovascular disease and the modified version contains 8 items and 2 subscales [12]. Responses could be given on a 5-point Likert scale (eg, “I know how to use the internet to find answers to my health-related questions,” 1=does not apply to me, 5=does apply to me).

#### eHealth-Related Data

The self-generated eHealth data questionnaire started with 3 items examining the participant’s general confidence in handling digital media, digital devices, and web-based platforms on the internet. They could respond on a 5-point Likert scale (1=not confident at all, 5=very confident). Patients were asked to self-assess their daily internet utilization period for private or work-related purposes. Moreover, internet anxiety was measured by 6 items with possible responses on a 5-point Likert scale (eg “I have concerns about using the internet,” 1=does not apply to me, 5=does apply to me). Finally, it was determined whether the participants were already experienced in using digital health interventions, had heard about them, or knew where to find them. The self-generated eHealth items had already been used in previous studies [13-16] and have proven good reliability.

### Statistical Analysis

We performed descriptive statistical analysis using SPSS (version 23; IBM Corp). Variables were presented as frequencies and percentages, as well as means and SDs. The participant’s BMI was calculated using the patient’s weight and height according to our hospital’s electronic medical records. Since it is often brought forward that older patients may not be a suitable target group for digital health interventions, we analyzed internet use frequency and digital confidence in relation to the participant’s age. To serve this purpose, we divided the participants into 4 age groups. To reach comparable sample sizes, we divided the age groups into <55, 55‐64, 65‐75, and >75-year-olds. Using these age groups, we performed an age-adjusted analysis of the variables “Internet-use,” as well as “confidence in handling internet platforms/digital media/mobile technologies.” This included an age-adjusted means comparison, using the Kruskal-Wallis test. We also conducted bivariate correlations between the participant’s age and the 4 items “Internet-use” and “confidence in handling Internet platforms/digital media/mobile technologies,” using Spearman’s Rho correlation for ordinally scaled variables. Finally, we analyzed these items mentioned above, describing the participants’ digital confidence adjusted to gender, and comparing the 2 formed subgroups “male” and “female.”

## Results

### Socioeconomic Characteristics

In total, 240 patients completed the assessment. With a mean age of 61.9 (SD 8.9) years and 17.4% (n=42) of participants being older than 70 years, the study showed an older people collective with a total of 59.9% (n=144) of all participants being male. With 60% (n=144) in total, most participants were married, 51.5% (n=124) of participants were retired, and 22.5% (n=54) of participants were working full-time.

### Comorbidities and Cardiovascular Risk Factors

An analysis of the prevalence of major cardiovascular risk factors showed that 79.6% (n=191) and 80.4% (n=193) of the study participants had hypercholesterinemia and arterial hypertension, respectively. One-fourth of the participants were active smokers (n=60, 25%) and 17% (n=41) had type-2 diabetes while 36.5% (n=88) of participants were overweight with a BMI of 25‐30 kg/m² and another 34.9% (n=84) of participants were obese with a BMI of >30 kg/m² (Table S1 in Multimedia Appendix 1). While all patients were diagnosed with coronary artery disease, a total of 36.7% (n=88) of patients had also been diagnosed with peripheral artery disease. Moreover, 26.3% (n=63) of participants had known congestive heart failure and 5.8% (n=14) of participants had once experienced a stroke (Table S1 in Multimedia Appendix 1).

### eHealth Data

#### Overview

Analyzing the frequency and duration of internet use per day, only 5.9% (n=14) of the study population did not use the internet daily and 53.4% (n=128) of participants stated an internet use of 1‐3 hours per day on average (Table 1). In general, levels of confidence when handling digital media, digital devices, and internet platforms were high in this study population (Table 2). Regarding internet-based programs to promote health or provide health information, only 34.4% (n=83) of participants knew how these programs worked and 47.5% (n=114) of participants knew where to find them (Table 3). When asked about having doubts about using the internet, a total of 75.7% (n=181) of the study population stated that this did not apply at all or rather did not apply. Furthermore, almost 80% (n=192) of the study population did not feel negatively affected by carrying a mobile phone with them (Table 4).

**Table 1.** Cardiovascular risk factors and comorbidities (N=240).

Cardiovascular risk factors	Prevalence, n (%)
BMI>30	84 (34.9)
BMI>25	88 (36.5)
Type 2 diabetes	41 (17)
Nicotine dependency	60 (25)
Hypercholesterolemia	191 (79.6)
Arterial hypertension	193 (80.4)
Congestive heart failure	63 (26.3)
Peripheral artery disease	88 (36.7)
Stroke	14 (5.8)

**Table 2.** Daily internet use (n=236).

	How long are you using the internet for private purposes per day?, n (%)
Not at all	14 (5.9)
Less than 1 hour	69 (29.2)
1‐3 hours	126 (53.4)
3‐5 hours	20 (8.5)
More than 5 hours	7 (3)

**Table 3.** Digital confidence in handling digital media (n=236).

	Very insecure, n (%)	Rather insecure, n (%)	Partly insecure, n (%)	Rather confident, n (%)	Very confident, n (%)
How confident are you when handling digital media?	16 (6.8)	17 (7.2)	56 (23.7)	92 (39)	55 (23.3)
How confident are you when handling internet platforms?	20 (8.5)	21 (8.9)	61 (25.8)	81 (34.3)	53 (22.5)
How confident are you when handling digital devices?	20 (8.5)	16 (6.8)	54 (22.9)	92 (39)	54 (22.9)

**Table 4.** Knowledge regarding internet-based programs to promote health (n=236).

	I do not agree at all, n (%)	I rather do not agree, n (%)	Neither, n (%)	I rather agree, n (%)	I fully agree, n (%)
I can imagine what that might be	27 (11.4)	45 (19.1)	35 (14.8)	97 (41.1)	32 (13.6)
I know how those programs work	36 (15.3)	66 (28)	53 (22.5)	67 (28.4)	14 (5.9)
I know where to find these programs	35 (14.8)	50 (21.2)	39 (16.5)	84 (35.6)	28 (11.9)

#### eHealth Literacy

As presented in Table S2 in Multimedia Appendix 1, the statistical analysis of the eHealth literacy questionnaire results showed that the majority (n=152, 63.5%) of the study participants rather or fully agreed to know how to find internet platforms with helpful information regarding their overall health. Moreover, a total of 71.2% (n=171) of participants stated to knew how to use the internet as a useful tool to get answers to their questions. When asked, whether the participants thought they were able to critically evaluate the credibility of health information on the internet, a total of 64% (n=152) of participants stated that they were able to do so. However, only 40% (n=96) of the participants stated to knew digital sources available for health information, and 47.9% (n=115) of participants felt secure enough to use information from the internet in order to make health-related decisions (Table 5).

**Table 5.** eHealth literacy (n=236).

	I do not agree at all, n (%)	I rather not agree, n (%)	Neither, n (%)	I rather agree, n (%)	I fully agree, n (%)
I know how to find internet platforms with helpful health information (n=236)	17 (7.2)	30 (12.7)	39 (16.5)	89 (37.7)	61 (25.8)
I know how to use the internet to get answers to my questions (n=236)	16 (6.8)	28 (11.9)	24 (10.2)	108 (45.8)	60 (25.4)
I know which sources for health information are available (n=235)	18 (7.7)	45 (19.1)	45 (19.1)	90 (38.3)	37 (1.7)
I know where I can find helpful health information on the internet (n=236)	18 (7.6)	32 (19.1)	39 (16.5)	103 (43.6)	44 (18.6)
I know how to use health information from the internet to help me (n=235)	24 (10.2)	48 (20.4)	50 (21.3)	88 (37.4)	25 (10.6)
I can critically evaluate health information on the internet (n=236)	17 (7.2)	23 (9.7)	45 (19.1)	101 (42.8)	50 (21.2)
I can distinguish between questionable and trustworthy internet resources with health information (n=236)	19 (8.1)	34 (14.4)	41 (17.4)	105 (44.5)	37 (15.7)
I feel secure to use information from the internet to make decisions regarding my health (n=236)	27 (11.4)	48 (20.3)	48 (20.3)	78 (33.1)	35 (14.8)

### Age-Related Differences in Internet Use and Digital Confidence

**Figure 1.**
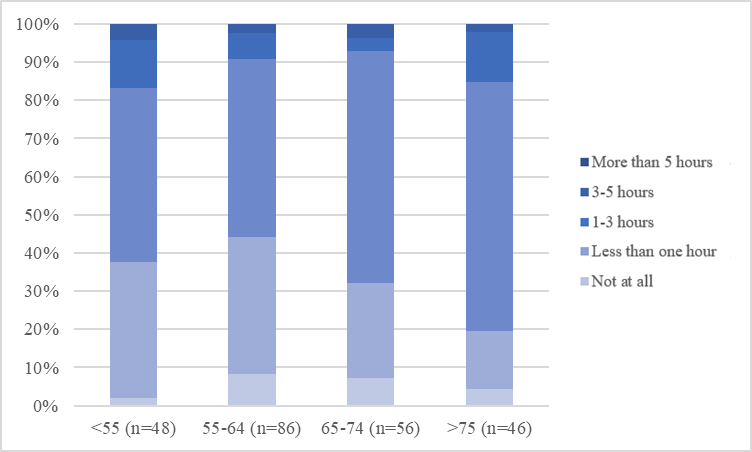
Daily internet use in relation to age groups (indicated in years along the x-axis).

**Figure 2.**
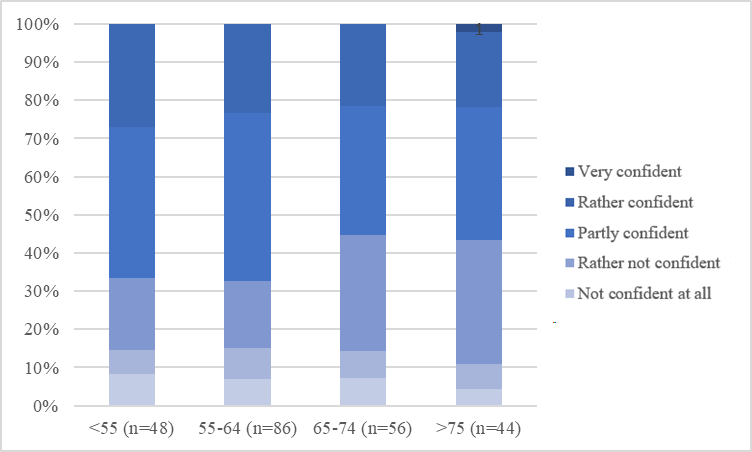
Confidence when handling digital media in relation to age groups (indicated in years along the x-axis).

**Figure 3.**
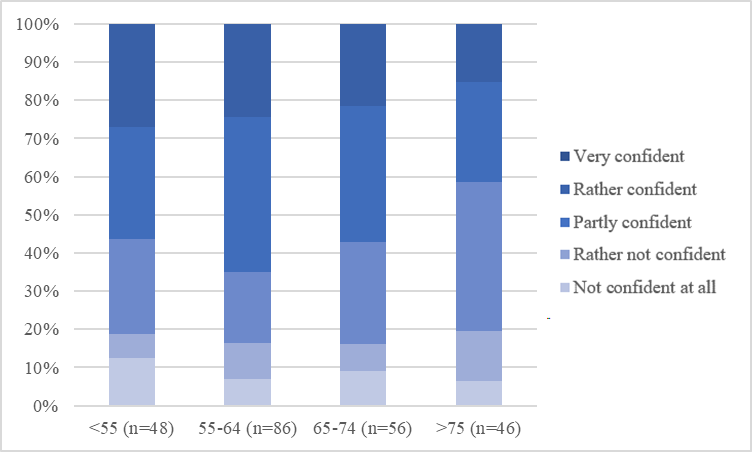
Confidence when handling digital devices in relation to age groups (indicated in years along the x-axis).

**Figure 4.**
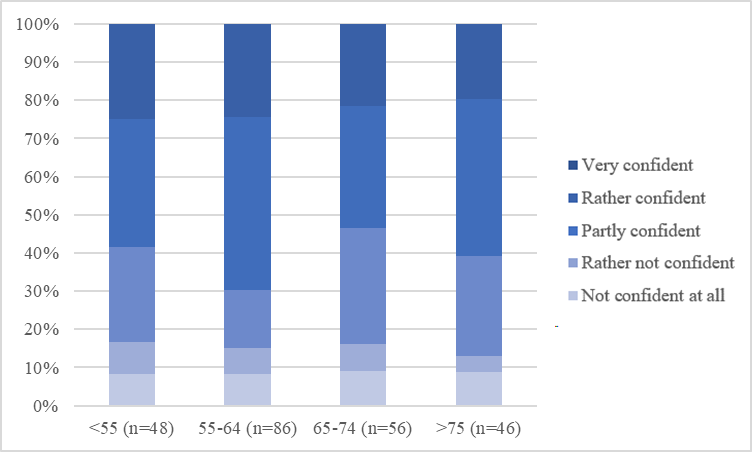
Confidence when handling internet platforms in relation to age groups (indicated in years along the x-axis).

As Figure 1 shows, using the internet for more than one hour a day, particularly for 1‐3 hours per day, was the most common answer when asked about the duration of daily internet use. This result can be seen throughout all age groups. A longer duration of daily internet use of at least 3 hours was more common in participants younger than 55 years than in older groups. When analyzing confidence in handling digital media, it becomes clear that with older age, starting from the age group of 65‐74-year-olds, the share of “partly confident,” “rather not confident,” and “not confident” individuals slightly increases, while still more than 50% (n=120) of the population remains “rather confident” or “very confident” (Figure 2). The share of at least “partly confident” participants is even higher in terms of confidence in handling digital devices such as smartphones and computers (Figure 3). This is again apparent throughout all age groups, even the oldest group of aged older than 75 years, contains less than 20% (n=48) of participants who are “not confident at all” in handling digital devices (Figure 3). Finally, confidence in handling internet platforms was generally lower than confidence in handling digital devices, and the share of participants who were “very confident,” as well as “rather confident,” decreased with older age. In the oldest age group of aged older than 75 years, 42% (n=101) of participants were “very confident” or “rather confident” in using internet platforms (Figure 4). Using the Kruskal Wallis test to compare internet use rates and digital confidence between the 4 age groups, there was no statistically significant difference for any of the examined items (>.05). Testing for correlations between the participants age and digital confidence, as well as daily internet use, the Spearman’s-Rho correlation shows the only statistically significant result regarding the confidence in using internet platforms (*ρ* [rho]) of –.128; *P*=.048). In addition, there was no age-related statistically significant difference in the levels of confidence in using mobile technologies (*ρ* of –0.70; *P*=.28), digital media (*ρ* of –0.96*; P*=.14), or daily internet use (*ρ* of 0.36*; P*=.58).

### Gender-Specific Differences in the Level of Digital Confidence

**Figure 5.**
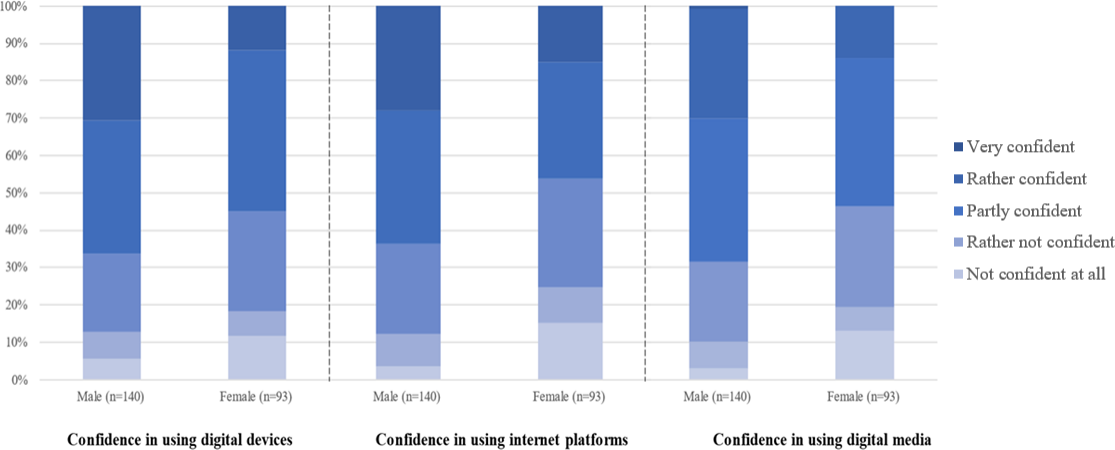
Gender-specific digital confidence.

As shown in Figure 5, we compared the differences in the levels of digital confidence in male and female participants. It is striking, that in all 3 categories, the levels of digital confidence seem to be at least slightly higher within the male subgroup. This difference becomes the most apparent comparing the levels of confidence when using internet platforms, with only about 10% (n=24) of the male participants feeling “rather not” or “not confident,” while about 25% (n=60) of the female participants feel that way. Moreover, the share of participants who stated to be “very confident” in either of the 3 subcategories is relevantly higher in the male patients.

## Discussion

### Internet Use and Digital Knowledge

We conducted an analysis of current internet use rates among individuals affected by cardiovascular disease, examining their attitudes toward the internet and mobile technology, with a specific focus on digital health interventions. Additionally, we assessed participants’ confidence in using digital technology and their ability to find credible health information on the internet.

Recent years have witnessed a notable increase in internet and smartphone use, particularly among the older population. From 2009 to 2019, the percentage of German citizens older than 65 years using the internet increased significantly, from approximately 30% to over 67% [17]. This trend was reflected in our trial, where only less than 6% (n=14) of the participants reported not using the internet for private purposes at all. Furthermore, the majority of the study collective (n=156, 64.9%) showed substantial daily internet use, spending at least one hour a day on the internet. Despite seemingly high internet use rates, knowledge about digital health interventions and internet-based health programs appeared to be limited. Only 34.3% (n=82) of participants indicated to understood how these programs worked, and merely 47.5% (n=114) of participants knew where to find them. This knowledge gap might be attributed to the potential lack of accessibility of digital interventions and a scarcity of programs that align with the specific needs of this patient demographic [18,19]. In the future, further research on patients’ specific concerns and barriers regarding implementing digital health interventions will be necessary to gain a more in-depth understanding of the current low utilization rates.

### Digital Confidence

Confidence levels in using digital media, internet platforms, and digital devices were notably high within this patient population, with over 60% (n=144) of participants expressing confidence in these 3 domains. Participants demonstrated particular assurance in handling digital media and devices. This heightened digital confidence is a crucial factor when contemplating the implementation of digital health interventions in this specific group, as a lack of confidence in digital technology is often cited as a significant barrier to integrating eHealth technologies into patients’ chronic disease management [20,21]. Notably, these results deviate from prior research, which has frequently indicated that older patients, in particular, harbored doubts about eHealth technologies and felt insecure about using them [8]. Given the mean age of 61.9 (SD 8.9) years of the study participants, the observed high levels of digital confidence may signify the rapidly increasing rates of smartphone ownership and internet use among the older population [17]. Supporting this hypothesis, even in age-adjusted analyses, participants displayed sustained high levels of digital confidence, with most individuals feeling assured in handling digital media and devices. Only a minority expressed a lack of confidence in using these technologies. However, to ensure the inclusion of these individuals and provide them with an opportunity to benefit from digital health interventions, additional education on the matter will be imperative.

### eHealth Literacy

To derive benefits from digital health solutions and effectively integrate them into daily life, possessing eHealth literacy is another crucial prerequisite [22]. Especially, in this study collective of patients with cardiovascular disease with a high prevalence of cardiovascular comorbidities and major risk factors, eHealth literacy is indispensable in order to use the rising possibilities that come with digitalization in chronic disease management.

eHealth literacy is defined as “the ability to seek, find, understand, and appraise health information from electronic sources and apply the knowledge gained to addressing or solving a health problem” [23]. It also involves the capability to discern between more or less credible sources of information. A common barrier to the adoption of digital health interventions is often the identification of trustworthy sources, leading to concerns about the security and privacy of these interventions [18,24]. In this trial, it was evident that most participants knew how to use the internet to find valuable health information and answers to health-related queries. However, it is noteworthy that only 40% (n=96) of the participants agreed that they knew about the various kinds of sources available for health information on the internet, with only 1.7% (n=4) fully agreeing. This lack of awareness could be a contributing factor to the low rates of prior engagement with digital health interventions. Previous studies have highlighted that the lack of knowledge about eHealth interventions remains a pertinent barrier, especially among older patients who may not be adequately informed about the diverse options available to receive health support [25]. The trial’s results indicated moderate levels of eHealth literacy. Most of the participants (n=154, 64%) felt capable of critically evaluating health information on the internet and distinguishing between questionable and trustworthy web-based resources. Nevertheless, only 47.9% (n=115) of the participants felt secure or reasonably secure in incorporating information from the internet into their health-related decision-making. Previous studies have indicated that even if health information is deemed credible, its implementation often falters due to the digital presentation, which is frequently described as “not user-friendly,” “not meaningful,” or generally challenging to comprehend [24]. One reason for this may be the sense of depersonalization of health information when presented digitally rather than in a face-to-face interaction, making it harder for individuals to connect with and apply relevant information [20].

### Outlook—Challenges and Opportunities of Digital Health Interventions

To address patients’ concerns regarding the security and reliability of health information on the internet, Germany took a significant step toward the digitalization of the health care system in 2019 by introducing digital health applications (DiGA) into standard care and supporting their use with statutory health insurance funding [26]. The approval of a mHealth or eHealth technology as a DiGA is strictly regulated and quality assurance for both prescribing physicians and patients using the intervention has to be ensured on a high level [27]. As highlighted in this study, the challenge of finding credible sources for health information on the internet remains a major concern. Having clear criteria to assess the quality and validity of health information that can be found on the internet is crucial to use the full potential of today’s digital technology [28]. Confidence in using internet platforms was generally lower in this patient population compared to confidence in using digital devices (Figures3  4). Therefore, officially approved digital health interventions with proven health care benefits that can be prescribed by treating physicians present an opportunity to make them available to more patients and ensure the high quality and credibility of the provided health information. Although this approach appears promising, the level of acceptance by both patients and physicians still needs to be thoroughly evaluated [29].

### Limitations

It should be noted that the eHealth literacy score was self-assessed, and this study did not try to match the perceived eHealth literacy score to the actual abilities to use the internet to find health information and implement it in daily life. It has been shown that self-assessed eHealth literacy scores and actual eHealth literacy can differ when put into practice [30,31]. Digital use patterns and internet use were also assessed using self-reported data, which makes them subject to potential bias. For future research, it could be beneficial to include tracking individuals’ daily use patterns in order to avoid potential bias and inaccuracies.

Moreover, since the study population was actively interested in participating in a trial revolving around mHealth technologies, a possible sampling bias should be considered when transferring the results to the general population. In addition to this, this trial particularly included patients diagnosed with coronary artery disease, which leads to a rather homogenous collective. This may limit the generalizability of these findings to a collective of patients with different cardiovascular diseases, such as heart failure, which was only present in one-fourth of the participants of this trial.

### Conclusions

This study revealed that individuals with atherosclerotic cardiovascular disease were generally well-acquainted with using the internet and handling digital devices in all age groups. The internet was an integral part of their daily lives, leading to a high level of confidence in digital technologies. However, the adoption of digital health interventions remained relatively low, possibly reflecting reservations about incorporating internet-derived information into daily routines. This hesitancy might stem from uncertainties in identifying credible and trustworthy health sources on the internet, as well as the perceived lack of personalization in digital health interventions.

Despite their older age, the results suggest that patients with cardiovascular disease can be a suitable target group for digital health interventions, given their high internet use rates and digital confidence. The patient population appears representative in terms of cardiovascular comorbidities and the prevalence of cardiovascular risk factors. To address persistent skepticism toward digital technologies, it becomes crucial to incorporate personalized health information and adopt a patient-centered approach. Therefore, we need to delve into the specific needs and requirements of our patient collective regarding digital health interventions. This approach is essential to enhance the acceptance and effectiveness of digital health interventions within this patient population.

## Supplementary material

10.2196/63057 Multimedia Appendix 1 Supplementary tables.

## Abbreviations

mHealth: mobile health
