# Supplementary material for: Digital Health Literacy and Attitudes Toward eHealth Technologies Among Patients With Cardiovascular Disease and Their Implications for Secondary Prevention: Survey Study
Source: JMIR Form Res. 2025 Mar 19;9:e63057. doi: 10.2196/63057 (PMC11939022; doi:10.2196/63057)
Supplement: Multimedia Appendix 1 [file formative-v9-e63057-s001.docx]

Table S1. Sociodemographic data (N=240).

|  | **Prevalence, n (%)** |
| --- | --- |
| Female | 96 (40.1) |
| Married | 142 (59.2) |
| Working full time | 53 (22.1) |
| Retired | 121 (50.4) |
| Incapacitated for work | 40 (16.7) |

Table S2. Burden through using the internet (n=235).

|  | **Does not apply, n (%)** | **Does rather not apply, n (%)** | **Partly applies, n (%)** | **Rather applies, n (%)** | **Fully applies, n (%)** |
| --- | --- | --- | --- | --- | --- |
| I do not feel well when I am constantly carrying a mobile phone | 127 (54) | 55 (23.4) | 28 (11.9) | 20 (8.5) | 5 (2.1) |
| I have doubts about using the internet | 141 (60) | 37 (15.7) | 37 (15.7) | 11 (4.6) | 9 (3.8) |
